# Exosomes and their derivatives as biomarkers and therapeutic delivery agents for cardiovascular diseases: Situations and challenges

**DOI:** 10.2478/jtim-2023-0124

**Published:** 2023-12-20

**Authors:** Yunyang Xu, Weimin Wan, Huixuan Zeng, Ze Xiang, Mo Li, Yiwen Yao, Yuan Li, Mariza Bortolanza, Jian Wu

**Affiliations:** Zhejiang University School of Medicine, Hangzhou 310009, Zhejiang Province, China; Department of Clinical Laboratory, The Affiliated Suzhou Hospital of Nanjing Medical University, Suzhou Municipal Hospital, Gusu School, Nanjing Medical University, Suzhou 215008, Jiangsu Province, China; Department of Internal Medicine V-Pulmonology, Allergology, Respiratory Intensive Care Medicine, Saarland University Hospital, 66424 Homburg, Germany; Department of Cardiology, The Affiliated Suzhou Hospital of Nanjing Medical University, Suzhou Municipal Hospital, Gusu School, Nanjing Medical University, Suzhou 215008, Jiangsu Province, China

**Keywords:** cardiovascular disease, exosome, miRNA, diagnosis, prognosis

## Abstract

Microvesicles known as exosomes have a diameter of 40 to 160 nm and are derived from small endosomal membranes. Exosomes have attracted increasing attention over the past ten years in part because they are functional vehicles that can deliver a variety of lipids, proteins, and nucleic acids to the target cells they encounter. Because of this function, exosomes may be used for the diagnosis, prognosis and treatment of many diseases. All throughout the world, cardiovascular diseases (CVDs) continue to be a significant cause of death. Because exosomes are mediators of communication between cells, which contribute to many physiological and pathological aspects, they may aid in improving CVD therapies as biomarkers for diagnosing and predicting CVDs. Many studies demonstrated that exosomes are associated with CVDs, such as coronary artery disease, heart failure, cardiomyopathy and atrial fibrillation. Exosomes participate in the progression or inhibition of these diseases mainly through the contents they deliver. However, the application of exosomes in diferent CVDs is not very mature. So further research is needed in this field.

## Introduction

All cells and most organisms produce physiologically functional extracellular vesicles (EVs). The classification of EVs is constantly changing, but they can generally be categorized into exosomes and ectosomes.^[1, 2, 3]^ Ectosomes are vesicles produced directly from the plasma membrane to the outgoing bud, which produces microvesicles, microparticles, and large vesicles diameters of 50 nm to 1 μm.^[1]^ In contrast, exosomes are small endosomederived membrane microvesicles with diameters of 40 to 160 nm.^[1,4]^ Over the past decade, increasing attention has been given to exosomes, partly because they are functional vehicles that carry a variety of lipids, proteins and nucleic acids ^[5, 6, 7, 8]^ to deliver to the surrounding cells. Moreover, there are also many important molecules on the exosomal membrane, such as tetraspanins (CD9, CD63, etc.), intracellular adhesion molecules (ICAMs), major histocompatibility complex (MHC) class I, MHC class II, integrins and different kinds of receptors, which play significant roles ^[4,9]^ by modifying target cells’ physiological functions. For instance, exosomes can establish intercellular communication by delivering cargo to recipient cells, which is essential for various cellular functions, including immune response, signal transduction, and antigen presentation.^[10-12]^ Furthermore, exosomes have enormous therapeutic potential for several of conditions, including chronic inflammation, lipid metabolism disorders, and cardiovascular disease, owing to their ability to transport biological material.^[13-15]^ Exosomes may also participate in numerous pathological processes in addition to their beneficial physiological roles. Certain exosome-borne microRNAs (miRNAs) are responsible for different cardiac diseases, such as stroke and hypertension.^[16-18]^

Cardiovascular diseases (CVDs), including hypertension, heart failure (HF), atherosclerosis, stroke, ischemic heart diseases, etc., continue to be a significant cause of death worldwide.^[14,19,20]^ It is predicted that the increasing incidence of CVDs will lead to more than 23.6 million deaths by 2030 globally.^[21,22]^ Atherosclerosis is the major cause of CVD, and its physiopathology also includes the remodeling of blood vessels, which may cause blood flow restrictions and influence the cardiovascular system.^[23]^ Numerous risk factors for CVD have been identified, such as diabetes, cigarette smoking, obesity, sedentary behavior, and unhealthy lifestyles. Moreover, genetic factors and aging are also significant risk factors that increases CVD prevalence.^[24]^ Studies have shown that CVDs are more common in patients with coronavirus illness 2019, who are at a higher risk of mortality.^[25-27]^ In summary, improving the rate of CVD recovery is critical.

Since exosomes are mediators of cell-to-cell communication, which are involved in several physiological and pathological processes and have the potential to function as biomarkers in clinical practice, they can play an integral role in improving CVD treatment.^[28]^ In particular, the possible application of exosomes as messengers or regenerative tools in CVDs has been actively investigated.^[29,30]^ In this review, we will introduce exosomes and their derivatives and discuss their existing and potential application in the diagnosis, prognosis and treatment of CVDs.

## Exosomes and their derivatives

Following the combination of late endosomes/multivesicular bodies (MVBs) with the plasma membrane, some vesicles are delivered to the outside of cells, and are referred to as exosomes, which were first detected in 1983.^[31]^ In 1985, this process was observed again in sheep.^[32]^ In 1987, Rose Johnstone chose the term “exosome” to nominate these vesicles.^[33]^ Initially, exosomes were viewed as waste products of damaged cells or byproducts without a function. Only in recent years has it been discovered that exosomes play an essential role in intercellular communication and many other cellular processes. Based on whether or not they have been artificially modified, exosomes can be divided into natural exosomes and engineered exosomes.^[34]^

### Exosome biogenesis

Three processes are involved in exosome biogenesis: the formation of early endosomes, growth of MVBs, and release of exosomes.^[35]^ The endosomes are formed after the invagination of the cell membrane followed by the accumulation of bioactive substances like proteins and miRNAs in the early sorting endosomes. Early endosomes then mature into late sorting endosomes (LSEs) with the participation of proteins such as the endocytosis sorting complex. After a second indentation, LSEs transform into MVBs. Eventually, MVBs fuse with the cell membrane and then the substances within MVBs are released from the cells in the form of vesicles, which are exosomes. The mechanisms for the formation of exosomes are diversified. One of the most well-known mechanisms is that this process depends on the endosomal sorting complexes required for transport (ESCRT) complexes.^[36,37]^ ESCRT combines with successive complexes on the MVB membranes and sorts cargos that are ultimately secreted as exosomes.^[1,38]^ In addition to this ESCRT-dependent mechanism, there are some ESCRT-independent mechanisms as well. For example, a mechanism associated with ceramides has been proposed, which is independent of ESCRT. The delivery of proteolipid protein into exosomes depends on the sphingolipid ceramide, and ESCRT is not involved in this process.^[39]^ Moreover, substances like four-transmembrane domain proteins and lipid rafts participate in the formation of some exosomes as well.^[40,41]^ In summary, exosome formation is complicated. The exact mechanism remains controversial and requires further research.

At the end of the formation process, exosomes detach from the plasma membrane and prepare for secretion, which can be categorized into autocrine, paracrine, and endocrine.^[42]^ The secretion of exosomes is also a complicated process, regulated by many factors. Exosome release involves being shed from the membrane; additionally, actin and myosin interaction and an ATP-dependent contraction are also indispensable.^[43]^ Many kinds of stimuli such as stress can also induce the secretion of exosomes.^[44]^ Moreover, exosome release can be governed by Rab-related proteins (Rabs) as well. Rab5 and Rab7 are essential for the delivery of cargo to early endosomes.^[45]^ Some other Rabs, such as Rab11 and Rab35, can also regulate exosome secretion.^[42]^ Additionally, soluble N-ethylmaleimide-sensitive factor attachment protein receptors (SNAREs), which promote vesicle fusion with the target membrane, can also regulate the secretion of exosomes.^[46]^ Bonifacino *et al*. have found that SNARE protein vesicle-associated membrane protein 7 (VAMP7) is essential for exosome delivery in leukemic cells.^[47]^ Another SNARE protein, YKT6, also regulates exosome release. Exosomal proteins, tumor susceptibility gene 101 protein (TSG101), WNT3A and vacuolar protein sorting-associated protein (VPS)26/35 released by human embryonic kidney HEK293 cells were reduced by YKT6 depletion, according to a study.^[48]^

**Figure 1:**
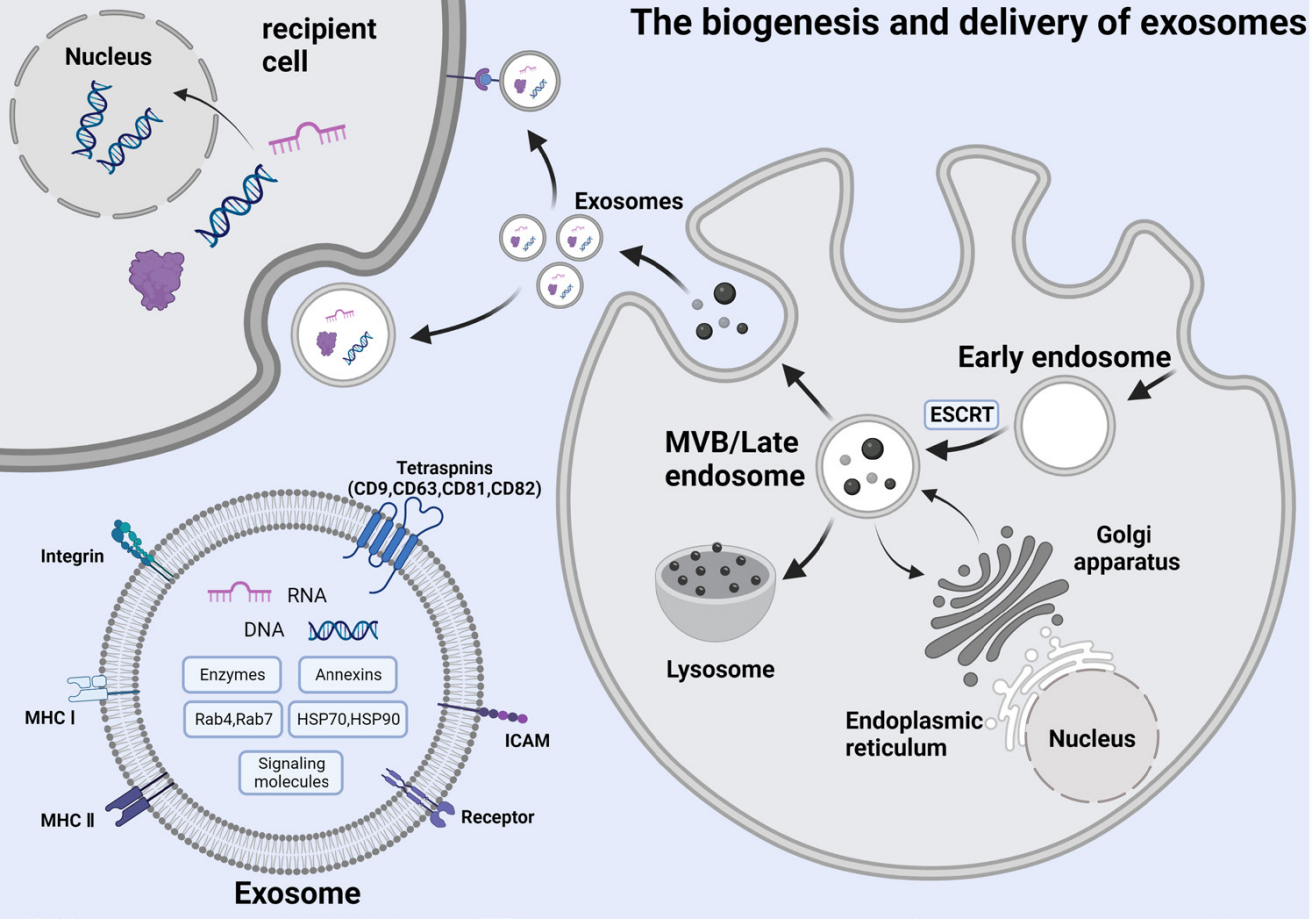
The biogenesis and delivery of exosomes.

### Physiological functions

Once exosomes exit the cells, they begin to carry out their physiological functions. Once released, exosomes fuse with recipient cells’ plasma membranes, releasing their contents.^[49]^ Surface proteins on some exosomes can bind to recipient cells’ surface receptors and induce intracellular signaling.^[50,51]^ After being absorbed, exosomal content can generate several physiological or pathological responses in recipient cells, one of the most critical functions being to induce an immune response. In dendritic cells (DCs), exosomes can deliver MHC class I and II molecules and then activate immune cells, such as CD8^+^ and CD4^+^ T-cells.^[52]^ Regardless of maturity, exosomes released by human DCs can promote a T helper 1 response.^[53]^ During bacterial infection, antibacterial immune responses can also be boosted by exosomes that promote the presentation of macrophage-derived bacterial antigens.^[54]^ Other physiological functions regulated by exosomes, such as stem cell maintenance and tissue repair, are all of great importance to the body.^[55,56]^

### Pathological functions

Besides biological functions, exosomes also have many pathological functions, for exosomes are involved in many diseases.^[57, 58, 59]^ As an example of neurodegenerative diseases, some studies have revealed that several proteins associated with neurodegenerative diseases are transported in exosomes. In patients with Parkinson’s disease (PD), Alzheimer’s disease (AD) and undrafted amyotrophic lateral sclerosis (ALS), exosomes containing aggregation-prone proteins were discovered.^[60-62]^ Exosomes are also involved in cancer. Several studies have previously reported that oncogenic proteins and nucleic acids can be delivered via exosomes into cancer cells, which can alter the activity of recipient cells and play a critical role in many physiological processes. Proteins, mRNAs and miRNAs from prostate cancer cell-derived exosomes can induce the neoplastic transformation of adipose-derived stem cells (ASCs). Additionally, other tumor-derived exosomes may boost tumor thrombosis and angiogenesis by activating endothelial cells.^[50,63]^ In CVDs, exosomes can play a significant role as well, including peripartum cardiomyopathy and sepsis-induced cardiomyopathy. Some researchers recently demonstrated that exosomes released by cardiac fibroblasts, which are enriched in miRNAs, can induce cardiomyocyte hypertrophy. This process is regulated by silencing sarcoplasmic protein sorbin, SH3 domain-containing protein 2 (SORBS2), PDZ and LIM domain 5 (PDLIM5) proteins^[64]^ (Figure 1).

Due to exosomes’ pathological or physiological functions, they can be applied in the diagnosis, prognosis, and treatment of numerous diseases. As exosomes contain many bioactive substances and can interact with their recipient cells, they may function as an appealing marker and treatment strategy in cancer and other pathologies. Besides, exosomes can carry medicinal drugs for corresponding diseases so that they are capable of delivering drugs with high drug-loading capacity and accuracy and poor immunogenicity. There is no doubt that exosomes can be a promising tool and provide new therapeutic approaches for many diseases,^[65-67]^ but further research is still required until they can be widely used in clinical practice.

## Exosomes and their derivatives in different cardiovascular diseases

As mentioned earlier, CVDs constitute the most common cause of death. The types and genetic diversity of CVDs, as well as the side effects of traditional gene therapy, make non-targeted therapies unsuitable for CVDs.^[68]^ Thus, there is a need to develop disease-specific therapies, such as the use of delivery vehicles with specific therapeutic targets. Exosomes may be a promising treatment strategy for CVDs.^[69-72]^ As a deliverer of the signaling molecules, exosome-regulated communication between different cells of the heart has been shown to play a crucial role in the progression of cardiac diseases and the maintenance of cardiac homeostasis.^[73]^ Here, we introduce the application of exosomes in a few different kinds of CVDs.

### Coronary artery diseases

Coronary artery disease (CAD), a kind of cardiovascular disease that is caused by atherosclerosis or occlusion of the coronary arteries, has been one of the major public health threats worldwide, especially in middle-aged and older individuals.^[74]^ The various clinical manifestations of CAD include myocardial infarction, stable and unstable angina, and sudden cardiac death.^[75]^ Furthermore, CAD can be divided into two categories, acute coronary syndrome (ACS) and stable CAD (SCAD), according to different clinical characteristics and therapeutic principles.

Because of exosomes’ essential roles in intercellular communication through the transfer of cargo, they can participate in the diagnosis, prognosis and treatment of CADs (Table 1). Especially the various RNAs wrapped in exosomes play very significant roles. Long noncoding RNAs (lncRNAs), which are more than 200 nucleotides in length, play a significant regulatory role in several diseases’ pathological processes.^[76]^ Studies have shown that some lncRNAs in exosomes are essential in the pathogenesis of CAD. In addition, lncRNAs may function as biomarkers and be a part of specific therapeutic strategies for CAD.^[77,78]^ For example, the death of macrophages and vascular endothelial cells in atherosclerosis is regulated by exosomal lncRNA growth arrest-specific 5 (GAS5).^[79]^ In another study, quantitative real-time reverse transcription-polymerase chain reaction (qRT-PCR) and microarray analysis verified the downregulation of SOCS2-AS1in patients with CADs. Since there is a clear link between exosomal SOCS2-AS1 and CAD morbidity, Liang *et al*. suggested that plasma exosome-derived SOCS2-AS1 may be a CAD indicator.^[80]^

**Table 1:** The application of exosomal contents in CAD

Exosomal content	Role	Mechanism	Type of study	References
lncRNA GAS5	Diagnosis and prognosis	Regulate vascular the endothelial apoptosis cells of macrophages and	*In vitro* study	[79]
lncRNA SOCS2-AS1	Diagnosis and prognosis	Be downregulated in plasma from CAD patients	Human study	[80]
miR-126	Diagnosis and prognosis	Be increased in patients with ACS	Human study	[81]
miR-21	Diagnosis and prognosis	Be downregulated in patients with ACS	Human study	[81]
PTEN	Diagnosis and prognosis	Be increased in patients with ACS	Human study	[81]
miR-208a	Diagnosis and prognosis	Be increased in patients with ACS	Human study	[82]
miR-146a	Diagnosis and prognosis	Be with increased inflammatory in patients response with ACS and associated	Human study	[83]
Cyr61	Diagnosis and prognosis	Be vascular increased remodeling in patients with ACS and regulate	Human study	[84]
miR-942-5p	Diagnosis and prognosis	Be increased in patients with SCAD and regulate VEGFA–VEGFR2, PI3K and MAPK signaling pathways	Human study	[89]
miR-149-5p	Diagnosis and prognosis	Be increased in patients with SCAD and regulate VEGFA–VEGFR2, PI3K and MAPK signaling pathways	Human study	[89]
miR-32-5p	Diagnosis and prognosis	Be increased in patients with SCAD and regulate VEGFA–VEGFR2, PI3K and MAPK signaling pathways	Human study	[89]
miR-19	Treatment	Inhibit OS by downregulating the expression of PTEN and BIM and activate the Akt and ERK signaling pathways	*In vitro* study	[96]
miR-210	Treatment	Increase by stimulating cardiac the progenitor PI3K/Akt cells’ pathway tolerance to OS	*In vitro* study	[98]
miR-133a	Treatment	Improve ejection cardiac fraction function and fractional through shortening left ventricular	*In vitro* study	[100]

ACS, one of the most common types of CADs, is a severe disease, which can be divided into unstable angina pectoris and acute myocardial infarction (AMI). Traditionally, clinically abnormal levels of certain cardiac biomarkers have been used to identify these diseases. Nowadays, some novel exosomal biomarkers have been identified for ACS diagnosis. Ling *et al*.^[81]^ demonstrated that the levels of miR-126, miR-21, and phosphatase and tensin homolog (PTEN) in serum exosomes were distinctly different in patients with ACS and healthy individuals. According to the study, the expression levels of miR-21 were down-regulated in the patients, whereas those of miR-126 and PTEN levels were higher. The results showed that miR-21, miR-126 and PTEN may serve as specific serum markers for ACS. Another miRNA, miR-208a, is also associated with ACS. Bi *et al*. detected that the exosomal miR-208a levels in patients with ACS were up-regulated, indicating the diagnostic and prognostic function of exosomal miR-208a for ACS.^[82]^ Furthermore, Li *et al*. found significantly increased levels of serum exosomal miR-146a in patients with ACS than in the control group, suggesting that it may be a novel diagnostic biomarker for such patients. The relationship between miR-146a and inflammatory response was also demonstrated in this study.^[83]^ Additionally, some exosomal proteins may be involved in the diagnosis and prognosis of ASC. Li *et al*. confirmed increased expression levels of exosome‑derived cysteine‑rich protein 61 (Cyr61) in the plasma and linked it to ASC in their study. This indicates that exosomal Cyr61 has the potential to be used in the diagnosis and prognosis of ACS. Moreover, Cyr61 can serve a regulatory role in vascular remodeling *in vitro*.^[84]^

When it comes to SCAD, exosomal miRNAs may also function as biomarkers. In a study, three circulating exosomal miRNAs, miR-942-5p, miR-149-5p, and miR-32-5p, were significantly increased in patients with SCAD, and atherosclerosis was the most significant pathological basis of CAD. Atherosclerosis is associated with important signaling pathways, such as the vascular endothelial growth factor A-vascular endothelial growth factor receptor 2 (VEGFA-VEGFR2) signaling pathway. These signaling pathways can generate diverse responses in the development of atherosclerosis as well as participate in the regulation of atherosclerosis.^[85-88]^ Through the regulation of these signaling pathways, the aforementioned three miRNAs may play essential roles in SCAD morbidity. They may also function as novel diagnostic and prognostic indicators for patients with SCAD owing to their high expression in such patients.^[89]^ Other than miRNAs, circular RNAs (circRNAs) are also abundant in exosomes, and may also function as prediction targets and biomarkers for CAD prognosis.^[90,91]^

In addition to their significant roles in CAD prognosis and diagnosis, exosomes can also be used in treatment. The progression of CADs, particularly AMI, is significantly influenced by oxidative stress (OS).^[92]^ Inequality between the antioxidant defense systems and the production of reactive oxygen species (ROS) is the leading cause of OS.^[93,94]^ Some studies have found that exosomes can regulate OS in CADs, and miRNAs play an important role in this process. An important factor in ischemia-reperfusion injury is OS-induced apoptosis following myocardial ischemiareperfusion, which was substantially reduced when miR-19a was injected into the mouse myocardium.^[95]^ The underlying mechanism involves miR-19a down-regulating the expression of PTEN and Bcl-2-like protein 11 (BIM) in cardiomyocytes and the activation of Akt and extracellular signal-regulated kinase (ERK) pathways.^[96]^ Transcriptional factor hypoxia-inducible factor (HIF-1) is crucial in the cellular response to hypoxic environments.^[97]^ Exosomal miR-210 can be up-regulated by HIF-1. Huang *et al*. found that the activation of the phosphoinositide 3-kinase (PI3K)/Akt pathway led to exosome-rich miR-210 in endothelial cells exposed to hypoxia and boost the cardiac progenitor cells’ tolerance to OS.^[98]^ Therefore, exosomal miR-210 may also be used in the treatment of CADs. miR-133a is a kind of miRNA abundant in the heart and can promote this organ’s growth and development.^[99]^ In the myocardial infarction (MI) rat models, the decrease in left ventricular ejection fraction can improved cardiac function as a result of miR-133 overexpression;^[100]^ thus, miR-133a is an MI biomarker.^[101]^ Exosomes derived from mesenchymal stem cells (MSCs) play crucial roles in immune responses. Li *et al*. demonstrated that the miR-let7/high-mobility group AT-hook 2 (HMGA2)/nuclear factor-κB (NF-κB) pathway induced M2 macrophage polarization and regulated atherosclerosis in mice in an MSC-exosome study. Moreover, MSC-exosomes inhibited macrophage infiltration through the miR-let7/insulin-like growth factor 2 mRNA-binding protein 1 (IGF2BP1)/PTEN pathway. Based on the findings of this study, MSC-exosomes may be an effective strategy for preventing atherosclerosis as they may significantly reduce inflammation in atherosclerosis plaques.^[102]^ Cochain *et al*. also studied the interaction between exosomes and macrophages. Numerous functional phenotypes of macrophages were found in atherosclerotic plaques, which responded to the microenvironment and played various roles in vascular inflammation and atherosclerosis.^[103]^ According to the findings of the research, the communication between cardiomyocytes and macrophages by promoting exosome-mediated macrophage polarization is critical for the maintenance of cardiac homeostasis. Exosomes may thus be a potential novel therapeutic strategy for several CADs by modulating macrophage polarization profiles.^[104]^

### Heart failure

Many patients with CVDs experience HF, which is a multifaceted and challenging condition. HF can be divided into systolic HF with reduced ejection fraction (HFrEF) and diastolic HF with preserved ejection fraction (HFpEF). Owing to the high morbidity and death rate, HF is a significant healthcare burden worldwide.^[105-108]^ Symptoms of HF include paroxysmal nocturnal dyspnea, orthopnea, and dyspnea. Furthermore, an insufficient heart rate can result in fatigue, weakness, and exercise intolerance. Because these symptoms are mainly nonspecific, it is difficult to distinguish patients with HF from those with other CVDs.^[109]^ Hence, the clinical diagnosis of HF is challenging and the difficulties in the diagnosis may result in delayed therapy, which will eventually result in an incorrect prognosis. It is necessary to identify molecules closely related to the occurrence and development of HF, since they may promote early detection.

Exosomes may participate in the clinical diagnosis, prognosis, and treatment of HF and resolve the challenges in determining HF (Table 2). One of the most prevalent types of cardiomyopathies, dilated cardiomyopathy (DCM), is the primary cause of HF.^[110]^ Acute HF (AHF) is a severe pathophysiological state of DCM. Wu *et al*. found that miR-92b-5p in serum exosomes was significantly up-regulated in patients with DCM-AHF. Additionally, it was demonstrated that exosomal miR-92b-5p was responsible for the progression of DCM-AHF owing to a positive connection between exosomal miR-92b-5p and cardiac chamber enlargement. Thus, serum exosomal miR-92b-5p is a valuable diagnostic and prognostic biomarker for DCM-AHF.^[111]^ Peripartum cardiomyopathy (PCM) is a severe pregnancy-correlated cardiomyopathy in women, which can lead to PCM-HF. The symptom of this disease is an abrupt occurrence of HF in the late stage of pregnancy.^[112]^ Damaged cardiomyocyte cells secrete miR-146a, which can be detected in serum exosomes and exosomes in circulation.^[113]^ Compared to those with DCM-AHF and pregnancy-matched healthy controls, patients with PCM-AHF experience an increase in the plasma level of miR-146a.^[114]^ Beg *et al*. also reported significantly higher levels of exosomal miR-146a in patients with HF than in healthy controls.^[113]^ Therefore, exosomal miR-146a in exosomes can be developed as a specific diagnostic and prognostic biomarker for PCM-AHF.^[115]^ Furthermore, exosomal miR-181c and miR-495 may be novel biomarkers for the pathogenesis of chronic HF (CHF).^[116]^ Exosomes exclusively contain high levels of hsa_circ_0097435, a type of circular RNA. Han *et al*. performed the analysis of exosomes obtained from peripheral blood samples in patients with HF and healthy individuals and showed upregulated levels of hsa_circ_0097435 in patients with HF, and hsa_circ_0097435 was found to play an essential role in HF progression. Thus, hsa_circ_0097435 is a valuable biomarker for the clinical diagnosis and prognosis of HF.^[117]^

**Table 2:** The application of exosomal contents in HF

Exosomal content	Role	Mechanism	Type of study	References
miR-92b-5p	Diagnosis and prognosis	Be increased in patients with AHF	Human study	[111]
miR-146a	Diagnosis and prognosis	Be specifically increased in patients with PCM-AHF	Human study	[113]
miR-181c	Diagnosis and prognosis	Monitor the progression of CHF	*In vitro* study	[116]
miR-495	Diagnosis and prognosis	Monitor the progression of CHF	*In vitro* study	[116]
has_circ_0097435	Diagnosis and prognosis	Promote HF occurrence and development	Human study	[117]
miR-30d-5p	Treatment	Be the downregulated cardioprotective in role patients of miR-with 30HF d in and HF reduce	*In vivo* study	[118]
miR-126a-5p	Treatment	Be downregulated in patients with HF and decrease cardiac microvessel density and impair ventricular function	Human study	[120]
miR-214-3p	Treatment	accelerate therapeutic the target progression of CHF and be used as	*In vivo* study	[122]
let-7i-5p	Treatment	Accelerate therapeutic the target progression of CHF and be used as	*In vivo* study	[122]
let-7g-5p	Treatment	Accelerate therapeutic the target progression of CHF and be used as	*In vivo* study	[122]
miR-129-5p	Treatment	Protect necrosis the factor heart receptor from failure by targeting tumor	*In vitro* study	[123]
miR-320a	Treatment	Promote myocardial fibroblast proliferation	*In vitro* study	[124]
miR-1246	Treatment	Attenuated damage hypoxia-induced myocardial tissue	*In vitro* study	[125]

**Table 5:** The application of exosomal contents in myocarditis

Exosomal content	Role	Mechanism	Type of study	References
hsa-miR-146a-5p	Diagnosis and prognosis	Has FM patients higher expression levels in exosomes of pediatric	Human study	[151]
hsa-miR-23a-3p	Diagnosis and prognosis	Has FM patients higher expression levels in exosomes of pediatric	Human study	[151]
hsa-miR-27a-3p	Diagnosis and prognosis	Has FM patients higher expression levels in exosomes of pediatric	Human study	[151]
hsa-miR-155	Diagnosis and prognosis	Be differently expressed in FM patients	Human study	[152]
hsa-miR-320a	Diagnosis and prognosis	Be differently expressed in FM patients	Human study	[152]
Anti-CVB3 vaccine	Treatment	Enhance resistance to CVB3-induced viral myocarditis	*In vivo* study	[154]

Exosomes are associated with the treatment of HF as well. Exosomal miR-30d-5p and miR-126a-5p have been associated with diabetic HFpEF. The levels of exosomal miR-30d, which is known to be cardioprotective, are decreased in HF.^[118,119]^ Reduced level of miR-126a also contributes to HF by impairing ventricular function.^[120]^ Huang *et al*. proposed that miR-30d and miR-126a may be valuable markers and therapeutic targets for HFpEF in patients with diabetes.^[121]^ In another study, the researchers tested the effects of the three miRNAs, miR-214-3p, let-7i-5p, and let-7g-5p, on inflammation. These are circulating exosomal co-change miRNAs and can be found in the rostral ventrolateral medulla (RVLM) in patients with CHF. The results demonstrated that miR-214-3p worked as a pro-inflammatory mediator, whereas let-7g-5p and let-7i-5p inhibited inflammation. These three exosomal miRNAs, according to the research, may accelerate CHF progression by enhancing RVLM inflammation by facilitating communication between the periphery and center.^[122]^ Therefore, these three co-change miRNAs in exosomes may be used as therapeutic targets for CHF in the future. Yan *et al*. suggested that exosomal miR-129-5p from MSC exosomes inhibited HF by targeting tumor necrosis factor receptor-associated factor 3 (TRAF3) and the subsequent NF-kB signaling. This regulatory axis can be used in the treatment of HF.^[123]^ Another miRNA, miR-320a, was also associated with HF. Compared to healthy controls, serum exosomal miR‑320a and sST2 content were not only significantly increased in patients with CHF, but the levels also correlated with clinical CHF indices. miR-320a can promote myocardial fibroblast proliferation through the phosphatidylinositol-4,5-bisphosphate 3-kinase, catalytic subunit alpha (PIK3CA)/Akt/mammalian target of rapamycin (mTOR) signaling pathway. As a result, exosomal miR‑320a may function as a specific target for therapeutic strategies for CHF.^[124]^ Moreover, human umbilical cord mesenchymal stem cells (hucMSCs) release miR-1246 during CHF and serine protease 23 (PRSS23) is a specific target of miR-1246. Wang *et al*. found that exosomal miR-1246 from hucMSCs inhibited HF by targeting PRSS23. The possible underlying mechanism of miR- 1246-mediated reduction in myocardial tissue damage is by targeting PRSS23, which is followed by the inhibition of smooth muscle actin signaling. Therefore, miR-1246 may also be used for the treatment of HF.^[125]^

### Cardiomyopathy

Cardiomyopathy is a group of heterogeneous myocardial diseases. They can be caused by abnormalities in cardiac mechanical and electrical activities, which are manifested as inappropriate hypertrophy or expansion of the ventricle. Exosomes can participate in the diagnosis, prognosis and treatment of cardiomyopathy in many ways (Table 3).

**Table 3:** The application of exosomal contents in cardiomyopathy

Exosomal content	Role	Mechanism	Type of study	References
miR-1	Diagnosis and prognosis	Be increased in patients with DCM	*In* Human *vivo* study study	[127]
miR-208	Diagnosis and prognosis	Be increased in patients with DCM	*In* Human *vivo* study study	[127]
miR-499	Diagnosis and prognosis	Be increased in patients with DCM	*In* Human *vivo* study study	[127]
miR-19b-3p	Diagnosis and prognosis	Positively correlate with myocardial status	*In vivo* study	[128]
miR-181b-5p	Diagnosis and prognosis	Positively correlate with myocardial status	*In vivo* study	[128]
miR-126	Diagnosis and prognosis	Be increased in patients with DCM	*In vivo* study	[129]
miR-320	Diagnosis and prognosis	Be increased in patients with DCM	*In vivo* study	[129]
miR-19a	Treatment	Preserve size cardiac contractile function and reduce infarct	*In vitro* study	[96]
miR-29b	Treatment	Alleviate fibrosis and cardiomyocyte uncoupling in DCM	*In vivo* study	[130]
miR-455	Treatment	Alleviate fibrosis and cardiomyocyte uncoupling in DCM	*In vivo* study	[130]
miR-26a	Treatment	Prevent cardiomyopathy CKD-induced muscle wasting and attenuate	*In vivo* study	[133]
miR-155	Treatment	Mediate downregulating cardiomyocyte FoxO3a pyroptosis protein expression in uremic hearts by	*In vivo* study	[134]
miR-122	Treatment	Improve cardiac remodeling and metabolic profiles	Human study	[136]
miR-29a	Treatment	Regulate cardiac parameters in human subjects	Human study	[137]

In 1972, Rubler first described diabetic cardiomyopathy (DCM) as cardiac structural abnormalities without many other traditional cardiovascular diseases.^[126]^ Many exosomal miRNAs can participate in the diagnosis of DCM. Some myocardial miRNAs, including miR-1, miR-208, and miR-499, are abundant in circulating exosomes. The changes in their expression levels may have a significant impact on myocardial injury and the levels of their expression may be indicators of DCM.^[127]^ During the progression of DCM, miR-19b-3p and miR-181b-5p positively correlate with myocardial status in circulating exosomes. Therefore, their expression levels may also be used for the diagnosis and prognosis of DCM.^[128]^ Moreover, Wang *et al*. found both expressions of miR-126 and miR-320 were increased in patients with DCM. miR-320 can inhibit angiogenesis and promote the development of cardiac function, so it may also be used in the treatment of DCM.^[129]^

In the clinical treatment of cardiomyopathy, exosomes may also count. Yu *et al*. demonstrated that exosomes derived from MSCs abundant in GATA4 improved cardiac function and decreased infarct size. Hence, these exosomes may be used for the treatment of DCM. The possible mechanism is the inhibition of PTEN directly increases Akt and ERK activation and up-regulates miR-19a levels.^[96]^ In another study, Chaturvedi *et al*. confirmed that exercise brings up a down-regulation in the expression of the matrix metalloproteinase 9 (MMP9) by up-regulating the expression levels of miR-29b and miR-455 in exosomes from cardiomyocytes, thereby inhibiting fibrosis in DCM.^[130]^ Chronic kidney disease (CKD) may cause some CVDs such as uremic cardiomyopathy (UCM) and CKD-related diseases.^[131,132]^ Wang *et al*. demonstrated that the high expression level of miR-26a in muscle prevented muscle wasting and attenuated cardiomyopathy via exosome-mediated miR-26a delivery. Results of the study suggested that miR-26a could inhibit muscle wasting and UCM by increasing insulin sensitivity.^[133]^ miR-155 was also associated with UCM in another study. It was found that exosomal miR-155 plays an important role in mediating cardiomyocyte pyroptosis in uremic hearts, and this function is associated with the down-regulation of FoxO3a protein expression. These findings may provide novel insight into UCM and provide a novel therapeutic target for the disease.^[134]^ Obesity has emerged as a global epidemic in recent years and is intimately linked to an increasing morbidity of heart disease.^[135]^ Exosomal miR-122 was found to regulate mitochondrial function and cardiac remodeling during obesity-related cardiomyopathy. Mechanistically, through direct binding to mitochondrial protein ADP-ribosylation factor-like 2 (Arl-2), miR-122 regulated mitochondrial function, as well as cardiac function. Moreover, the researchers found circulating expression levels of miR-122 were positively correlated to the increase of NT-proBNP and negatively correlated to the cardiac ejection fraction (EF), and these two indicators are essential in indicating cardiomyopathy. Besides, hepatic miR-122 blocking could promote cardiac remodeling and metabolic profiles in mice with obesity and primary cardiomyocytes. All these findings indicated that miR-122 in exosomes might be used to treat obesity-related cardiomyopathy.^[136]^ Another exosomal miRNA, miR-29a, is also associated with obesity-related cardiomyopathy. Li *et al*. found there is a link between exosomal miR-29a and cardiac parameters in patients, and miR-29a sponge therapy can be used to combat obesity-mediated cardiac dysfunction. So, miR-29a may also play an important role in the clinical treatment of obesity-related cardiomyopathy.^[137]^ Besides, cardiac stem cells (CSCs) can be used for the therapies for cardiomyopathies, but some limitations exist. Most of CSCs’ regenerative effects are attributed to CSC-derived exosomes (CSC-XOs).^[138]^ In a study on CSC-XOs, Vandergriff *et al*. demonstrated that mice receiving CSC-XOs acquired superior cardiac function, as well as attenuated apoptosis and fibrosis. So, the application of CSC-XOs has the potential to overcome the limitations of existing therapies and develop cardiomyopathy treatment strategy.^[139]^

### Atrial fibrillation

Atrial fibrillation (AF) is the most prevalent one of heart arrhythmia and a significant worldwide burden.^[140]^ The pathogenesis of this disease is associated with the patients’ age and many other factors.^[141]^ Recent studies on AF have demonstrated the application of exosomes in the diagnosis, prognosis and treatment of AF (Table 4).

**Table 4:** The application of exosomal contents in AF

Exosomal content	Role	Mechanism	Type of study	References
miR-483-5p	Diagnosis and prognosis	Be correlated with the AF	Human study	[142]
miR-142-5p	Diagnosis and prognosis	Be correlated with the AF	Human study	[142]
miR-223-3p	Diagnosis and prognosis	Be correlated with the AF	Human study	[142]
miR-382-3p	Diagnosis and prognosis	Regulate the AF progression	Human study	[143]
miR-450a-2-3p	Diagnosis and prognosis	Regulate the AF progression	Human study	[143]
miR-3126-5p	Diagnosis and prognosis	Regulate the AF progression	Human study	[143]
miR-146	Treatment	Inhibit EGR1 myocardial fibrosis by down-regulating the gene	Human study	[144]
miR-17	Treatment	Inhibit TGF-β-induced fibrosis under oxidative stress	*In vitro* study	[145]
miR-210	Treatment	Inhibit TGF-β-induced fibrosis under oxidative stress	*In vitro* study	[145]
miR-320d	Treatment	Inhibit regulating cardiomyocyte STAT3 expression apoptosis in AF by negatively	*In vivo* study	[146]
miR-150-5p	Treatment	Reduce the immune reactions and suppress the Th1 proliferation and secretion of the pro-inflammatory cytokines	*In vitro* study	[147]
miR-142-3p	Treatment	Reduce the immune reactions and suppress the Th1 proliferation and secretion of the pro-inflammatory cytokines	*In vitro* study	[147]
Let-7d	Treatment	Reduce the immune reactions and suppress the Th1 proliferation and secretion of the pro-inflammatory cytokines	*In vitro* study	[147]

Many kinds of miRNAs in the exosomes can work as diagnostic biomarkers for AF. According to the research, the levels of miR-483-5p, miR-142-5p, and miR-223-3p were linked to AF, and further research demonstrated that the miR-483-5p was independently related to AF. Therefore these miRNAs may become specific diagnostic and prognostic biomarkers for AF.^[142]^ In another study, Liu *et al*. found that the miR-382-3p, miR-450a-2-3p, and miR−3126-5p in the exosomes are associated with AF, and especially the miR-382-3p seemed pivotal in the AF progression.^[143]^

Exosomes can also be used as therapeutic approaches in the treatment of AF. The pathogenesis of the AF is mainly owed to fibrosis, remodeling, inflammation, and apoptosis in the heart. As exosomes can specifically regulate these processes, they can play significant roles in the clinical therapies for AF. miR-146 in exosomes from Adipose-derived stem cells (ADSCs) can inhibit myocardial fibrosis. Therefore, this kind of miRNA may be essential in treating AF. The mechanism is miR-146 could prevent myocardial fibrosis by inhibiting the expression of the gene EGR1.^[144]^ Besides, exosomal miR-17 and miR-210 can also inhibit TGF- β-induced fibrosis.^[145]^ The exosomal miR-320d from the ADSCs can indirectly inhibit cardiomyocyte apoptosis in AF by negatively regulating STAT3 expression, thus increasing survival. This method may provide novel treatment strategies for AF, thereby improving the clinical treatment of AF.^[146]^ Furthermore, exosomes created from Tregs may transport the miR-150-5p, miR-142-3p, and Let-7d to dendritic cells (DCs) and T-helper 1 (Th1), reducing immune responses, and inhibiting the secretion of pro-inflammatory cytokines. These regulatory functions may be used to treat AF.^[147]^ Xu *et al*. found that the delivery of Lv-Nrf2 exosomes could suppress AF-induced arrhythmias, myocardial fibrosis, apoptosis, and inflammation. Therefore, this study provides a potential novel approach for the therapies for AF.^[148]^

### Myocarditis

Myocarditis is defined as an inflammatory process of the myocardium. It can result in cardiac dysfunctions, such as progressively deteriorated diastolic and systolic function or arrhythmias.^[149]^ The most severe form of myocarditis is fulminant myocarditis (FM), which develops rapidly and can lead to acute cardiac shocks.^[150]^

Exosomes play important roles in myocarditis as well. Zhang *et al*. verified higher expression levels of hsamiR-146a-5p, hsa-miR-23a-3p, and hsa-miR-27a-3p in the exosomes of pediatric patients with FM using qRT-PCR, indicating that these three miRNAs can be used for the diagnosis of FM.^[151]^ Another study determined that hsa-miR-155 and hsa-miR-320a provided an excellent diagnostic capability for patients with FM.^[152]^

Viral infection is a major cause of myocarditis. Coxsackievirus B3 (CVB3), an enterovirus, is believed to be the most common causative agent of myocarditis.^[153]^ Zhang *et al*. constructed an exosome-based anti-CVB3 vaccine (exo-VP1) that enhanced the resistance of mice to CVB3-induced viral myocarditis.^[154]^ Gu *et al*. found that hucMSC-exosomes alleviated CVB3-induced myocarditis by activating the 5’ AMP-activated protein kinase (AMPK)/mTOR-mediated autophagy flux pathway to attenuate cardiomyocyte apoptosis, which will be beneficial for MSC-exosome therapy of myocarditis.^[155]^

In summary, exosomes, especially exosomal miRNAs, are significant in these and possibly other CVDs. They may serve as a new strategy for diagnosing, treating and preventing CVDs in the future. For example, using exosomes as drug delivery vehicles is an important application of exosomes. In recent years, an increasing number of studies have revealed that exosomes could function as attractive biological vesicles for the delivery of biological therapeutics across different biological barriers to target cells ^[4, 156, 157]^. Compared to available treatments, this method is non-invasive and may be more effective for CVDs. In the future, using exosomal delivery of drugs to treat CVD may have very good application prospects.

However, this new strategy is in its infancy. There are still several hurdles before achieving clinical application.^[158,159]^ For example, the purification and separation technology of exosomes is not mature enough for clinical application.^[160]^ Furthermore, exosomes contain a variety of biologically active substances, some of which may help inhibit CVDs, whereas others may cause adverse or side effects in the cardiac tissue.

In our opinion, the researchers should focus on improving the purification and separation technology of exosomes, thus removing harmful substances and making exosomes more suitable for treatment. Exosome therapies remain at the research stage, and the practical clinical results and potential side effects are unknown. Whether exosomes can be widely applied in clinical practice requires further research.

## Conclusions

Exosomes have received increasing attention partly because they are functional vehicles that carry a variety of lipids, proteins and nucleic acids and can deliver these cargos to the target cells they encounter. Because of this function, exosomes may be used for the diagnosis, prognosis and treatment of many diseases, including CVDs. Because of the exosomes’ biological function, they may function as biomarkers for the diagnosis and prognosis of CVDs and improve the therapies for CVDs. Many studies have demonstrated that exosomes are associated with CVDs, such as coronary artery disease, heart failure, cardiomyopathy, and atrial fibrillation. Exosomes participate in the progression or inhibition of these diseases mainly through the contents they deliver, especially different kinds of micro RNAs. However, because the application of exosomes in different CVDs is still an immature field, further research is needed.
